# In Vitro Antimicrobial Activity of Five Newly Approved Antibiotics against Carbapenemase-Producing Enterobacteria—A Pilot Study in Bulgaria

**DOI:** 10.3390/antibiotics13010081

**Published:** 2024-01-15

**Authors:** Rumyana Markovska, Petya Stankova, Temenuga Stoeva, Emma Keuleyan, Kalina Mihova, Lyudmila Boyanova

**Affiliations:** 1 Department of Medical Microbiology, Medical Faculty, Medical University of Sofia, 1431 Sofia, Bulgaria; petya.stankova88@abv.bg (P.S.); l.boyanova@hotmail.com (L.B.); 2 Department of Microbiology and Virology, University Multiprofile Hospital for Active Treatment (UMHAT) ”Saint Marina”, Medical University of Varna, 9002 Varna, Bulgaria; temenuga.stoeva@abv.bg; 3 Department of Clinical Microbiology, Medical Institute-Ministry of the Interior, 1606 Sofia, Bulgaria; eekeuleyan@abv.bg; 4 Molecular Medicine Center, Medical University of Sofia, 1431 Sofia, Bulgaria; kmihova@mmcbg.org

**Keywords:** carbapenemases, *Enterobacterales*, novel antibiotics

## Abstract

To solve the problem with pan-drug resistant and extensively drug-resistant Gram-negative microbes, newly approved drugs such as ceftazidime/avibactam, cefiderocol, plazomicin, meropenem/vaborbactam, and eravacycline have been introduced in practice. The aim of the present study was to collect carbapenemase-producing clinical *Enterobacterales* isolates, to characterize their carbapenemase genes and clonal relatedness, and to detect their susceptibility to commonly used antimicrobials and the above-mentioned newly approved antibiotics. Sixty-four carbapenemase producers were collected in a period of one year from four Bulgarian hospitals, mainly including *Klebsiella pneumoniae* (89% of the isolates) and also single *Proteus mirabilis*, *Providencia stuartii* and *Citrobacter freundii* isolates. The main genotype was *bla*_NDM-1_ (in 61%), followed by *bla*_KPC-2_ (23%), *bla*_VIM-1_ (7.8%) and *bla*_OXA-48_ (7.8%). Many isolates showed the presence of ESBL (*bla*_CTX-M-15/-3_ in 76.6%) and AmpC (*bla*_CMY-4_ in 37.5% or *bla*_CMY-99_ in 7.8% of isolates). The most common MLST type was *K. pneumoniae* ST11 (57.8%), followed by ST340 (12.5%), ST258 (6.3%) and ST101 (6.3%). The isolates were highly resistant to standard-group antibiotics, except they were susceptible to tigecycline (83.1%), colistin (79.7%), fosfomycin (32.8%), and aminoglycosides (20.3–35.9%). Among the newly approved compounds, plazomicin (90.6%) and eravacycline (76.3%) showed the best activity. Susceptibility to ceftazidime/avibactam and meropenem/vaborbactam was 34.4% and 27.6%, respectively. For cefiderocol, a large discrepancy was observed between the percentages of susceptible isolates according to EUCAST susceptibility breakpoints (37.5%) and those of CLSI (71.8%), detected by the disk diffusion method. This study is the first report to show patterns of susceptibility to five newly approved antibiotics among molecularly characterized isolates in Bulgaria. The data may contribute to both the improvement of treatment of individual patients and the choice of infection control strategy and antibiotic policy.

## 1. Introduction

Globally, antimicrobial resistance is one of the major problems for health care systems nowadays. This leads to increased costs and destabilization of the health infrastructure. According to the European Centre for Disease Prevention and Control (ECDC), more than 35,000 people die due to antimicrobial-resistant infections in the European Union/European Economic Area (EU/EEA) each year [1]. Similarly, a recent report by the Center for Disease Control and Prevention (CDC) in 2019 stated that antimicrobial resistance is associated with over 35,000 deaths each year [2]. The increased frequency of carbapenem-resistant Gram-negative bacteria significantly contributes to the problem [3,4]. Carbapenem-resistant *Enterobacterales* (CRE) isolates remain a significant public health threat, causing a wide range of intrahospital bloodstream, respiratory, urinary tract, and intraabdominal infections [3,4]. In Europe, the incidence of carbapenem-resistant invasive isolates of *Klebsiella pneumoniae* dramatically increased from 2019 (baseline) to 2022 by 49.7% [5]; the same trend was observed in Bulgaria, where the frequency of these isolates increased from 0% in 2013 to 47.3% in 2022, with the biggest increase in the last two years [6]. The most important mechanism of carbapenem resistance is mediated by production of different carbapenemases, most of them destroying almost all carbapenems and other beta-lactams and demonstrating non-susceptibility to older beta-lactamase inhibitors such as sulbactam, clavulanic acid and tazobactam [3]. The class A *Klebsiella* producing carbapenemase (KPC), class D oxacillinases (OXA)-OXA-48, OXA-181 and their variants, as well as the class B metallo-beta-lactamases (MBLs) (New Delhi Metallo beta-lactamase, NDM; imipenemase, IMP; Verona Integron Metallo-carbapenemase, VIM) [3,7] are among the most common carbapenemases in *Enterobacterales*. During the last few years, clinically significant KPC-2, NDM-1, VIM and OXA-48 producing isolates of *Klebsiella pneumoniae* and OXA-23, OXA-24 and OXA-72 producing *Acinetobacter baumanii* have been detected in Bulgaria [8,9,10]. Taking into consideration these facts as well as the higher frequency of isolation of carbapenem-resistant invasive isolates of *K. pneumoniae* in 2019–2022 [5] and the appearance of pan-drug resistant isolates [9], as well the limited therapeutic options for them, new treatment alternatives are strongly needed [11]. In the last decade, several newly approved antibacterial agents, such as-ceftazidime/avibactam, cefiderocol, plazomicin, meropenem/vaborbactam and eravacycline, have been introduced into medical practice [12,13].

The aim of this study was to characterize the carbapenemase genes among carbapenem-resistant enterobacterial isolates, to evaluate their clonal relatedness, and to detect the in vitro susceptibility to commonly used antimicrobials and to five newly introduced agents (ceftazidime/avibactam, cefiderocol, plazomicin, meropenem/vaborbactam, eravacycline), as well as to assess the association between susceptibility and carbapenemase genotypes. 

## 2. Materials and Methods

### 2.1. Bacterial Isolates

Four hospitals located in three Bulgarian cities were included in this study: University Multiprofile Hospital for Active Treatment (UMHAT) “Saint Marina”, Varna, UMHAT “Georgi Stranski”, Pleven, UMHAT “Ivan Rilski”, Sofia, and Medical Institute-Ministry of the Interior, Sofia. During the period January–December 2018, non-duplicate (one per patient), clinically significant carbapenem non-susceptible isolates of the order *Enterobacterales* were collected from patients admitted to the four hospitals. Species identification was performed by VITEK 2 compact (bioMérieux, Marcy-l’Étoile, France) or Phoenix automated systems (Becton Dickinson, Springfield, IL, USA) and confirmed by matrix-assisted laser desorption/ionization time of flight mass spectrophotometry (MALDI TOF MS) (VITEK MS (bioMérieux, France); MALDI Biotyper Syrius (Bruker, Daltonics, Bremen, DE, USA)).

### 2.2. Phenotypic Carbapenemase Detection

A phenotypic confirmation of carbapenemase production was performed by the modified Hodge test and by the KPC&MBL&OXA-48 disk kit (Liofilchem, Roseto degli Abruzzi, Italy), acc. EUCAST, 2023.

### 2.3. PCR, Sequencing, MLST Typing

All isolates were screened for the presence of *bla*_VIM_, *bla*_IMP_, *bla*_KPC_, *bla*_NDM_, *bla*_OXA-48_ *bla*_CTX-M_, *bla*_CMY_, *bla*_FOX_, *bla*_DNA_ and *bla*_ACC_ genes, as previously described [9,14,15]. The genes were sequenced using primers binding outside the coding region of *bla*_CTX-M-1-group_, *bla*_KPC_ [5], *bla*_CMY_, *bla*_VIM_ [9] and *bla*_NDM_ [16]. The nucleotide and deduced amino acid sequences were analyzed, and multiple alignments were performed using Chromas Lite 2.01(Technelysium Pty Ltd., Brisbane, Australia) and DNAMAN version 8.0 Software (Lynnon BioSoft, Vaudreuil-Dorion, QC, Canada).

For *K. pneumoniae* species complex isolates, the MLST typing procedure and primers were used according to https://bigsdb.pasteur.fr/klebsiella/primers-used/ (accessed on 21 April 2023). The assignments to allelic numbers and sequence types (STs) were carried out as described in the MLST database (Pasteur Institute, Paris, France; https://bigsdb.pasteur.fr/klebsiella/, accessed on 21 April 2023). The protocols, primers and ST assessment for *Enterobacter cloacae* were carried out according to the MLST database (https://pubmlst.org/organisms/enterobacter-cloacae, accessed on 21 April 2023). A clonal complex was defined as a group of two or more independent isolates that shared six identical alleles. 

### 2.4. Susceptibility Testing

Susceptibility testing to a set of 16 antimicrobial agents was performed by Kirby-Bauer disk diffusion method on Müeller–Hinton II agar according to EUCAST guidelines (http://www.eucast.org/clinical_breakpoints/, access date 21 April 2023) [17] with disks containing amoxicillin-clavulanic acid (20/10 µg), piperacillin-tazobactam (30/6 µg), cefotaxime (5 µg), ceftazidime (10 µg), ceftazidime/avibactam (10/4 µg), cefepime (30 µg), cefoxitin (30 µg), imipenem (10 µg), meropenem (10 µg), cefiderocol (30 µg), tobramycin (10 µg), gentamicin (10 µg), amikacin (30 µg), trimethoprim/sulfamethoxazole (1.25/23.75), ciprofloxacin (5 µg), and levofloxacin (5 µg). Minimal inhibitory concentrations (MICs) of meropenem, tigecycline, meropenem/vaborbactam and eravacycline were determined by MIC test strips (Liofilchem, Italy), and for colistin, a broth microdilution method was used (SensiTest Colistin, Liofilchem, Italy). Fosfomycin agar dilution panel was used to determine the susceptibility to fosfomycin (Liofilchem, Italy). The results were interpreted according to EUCAST, 2023 v. 13.1 guidelines. Food and Drug Administration (FDA) breakpoints were used (≤2 mg/L for susceptibility, 4 mg/L for intermediate susceptibility, and ≥8 mg/L for resistance to tigecycline, as well as ≤0.5 mg/L for susceptibility and ≥1 mg/L for nonsusceptibility to eravacycline) [18,19]. For plazomicin, the recommendations of manufacturers/FDA for breakpoints were used [20] (≤2 mg/L for susceptibility, 4 mg/L for intermediate susceptibility and ≥8 mg/L for resistance). Multidrug-resistant (MDR) and extensively drug-resistant (XDR) isolates were determined following previously defined criteria—for MDR, if the isolates were non-susceptible to at least one agent in ≥3 antimicrobial groups, and XDR was defined when isolates were non-susceptible to at least one agent in all but two or fewer antimicrobial groups [21].

### 2.5. Statistical Analysis

Chi-square test and Fisher’s exact test of independence were performed to compare the variables of interest (https://www.graphpad.com/quickcalcs/contingency1/, accessed on 21 April 2023).

## 3. Results

### 3.1. Bacterial Isolates 

A total of 69 carbapenem non-susceptible isolates were collected from urine samples (*n* = 44), broncho-tracheal secretions (*n* = 17), wounds (*n* = 4) and blood cultures (*n* = 4). Thirty-nine isolates were obtained from ICU patients (56.5%). 

### 3.2. Phenotypic Carbapenemase Detection

The phenotypic tests determined 64 isolates (92.7%) as possible carbapenemase producers, including 44 isolates as probable metallo-carbapenemase producers; 15 as possible KPC producers; and 5 as likely OXA-48 producers. Among them, *K. pneumoniae* was the most frequently isolated bacterial species (89%, *n* = 57). *Proteus mirabilis* (*n* = 3), *Providentia stuartii* (*n* = 2), *Citrobacter freundii* (*n* = 2) and *Enterobacter cloacae* complex (*n* = 1) were less frequently detected. For the present study, we used these 64 carbapenemase-producing isolates. 

### 3.3. PCR, Sequencing, MLST Typing 

PCR and sequencing confirmed the results of the phenotypic detection and identified *bla*_NDM-1_ in 39 *K. pneumoniae* isolates (61%), *bla*_KPC-2_ in 15 isolates (23%) (*K pneumoniae*, *n* = 13; *C. freundii*, *n* = 2), *bla*_OXA-48_ in 5 isolates (7.8%) (*K. pneumoniae*, *n* = 4; *E. cloacae* complex, *n* = 1) and *bla*_VIM-1_ in 5 isolates (*P. mirabilis*, *n* = 3; *P. stuartii*, *n = 2*) (Table 1). Genes encoding CTX-M-15 and CTX-M-3 ESBLs were detected in 43 (67.2%) and 6 isolates (9.4%), respectively. Five isolates (*P. mirabilis*, *n* = 3; *P. stuartii*, *n* = 2) were positive for both *bla*_CMY-99_ (encoded AmpC enzymes) and *bla*_VIM-1._ *bla*_CMY-4_ was found in 24 isolates (37.5%). Co-production of NDM-1, CTX-M-15/-3 and CMY-4 beta-lactamases was observed in 22 isolates. All studied isolates were negative for *bla*_ACC_, *bla*_DHA_ and *bla*_FOX_.

MLST revealed four major clones of *K. pneumoniae* (ST11, ST340, ST101 and ST258) with the predominance of ST11 (57.8%) (Table 2), followed by ST340 (12.5%), ST258 (6.3%) and ST101 (6.3%). ST11, ST340 and ST258 were members of clonal complex CC258. The association between clones, carbapenemase genes, ESBLs, bacterial species and center of origin is shown in Table 2. Eight different genotype combinations were observed. The *bla*NDM-1 positive isolates were predominantly associated with ST11 clone, and the most common isolates were *bla*_NDM-1_ + *bla*_CTX-M-3/-15_ + *bla*_CMY-4_ belonging to the ST11 clone. Among 34 *bla*_CTX-M_, *bla*_NDM-1_ positive isolates, only four isolates showed presence of the *bla*_CTX-M-3_ gene; the other isolates produced CTX-M-15. ST258, ST340, and ST34 were associated with KPC-2, and ST101 with OXA-48. The single isolate *E cloacae* complex belonged to ST200 clone. 

### 3.4. Susceptibility Testing

The susceptibility testing demonstrated that 100% of the isolates were resistant/non-susceptible to amoxicillin-clavulanic acid, piperacillin-tazobactam, cefotaxime, ceftazidime, cefepime, imipenem, meropenem, tobramycin, trimethoprim/sulfamethoxazole, ciprofloxacin, and levofloxacin (Table 3). The resistance rate to cefoxitin was 96.9%. The susceptibility to aminoglycosides varied between 20.3% and 35.9%. Higher rates of susceptibility to gentamicin were detected among the MBL producers (46.7%), (*p* = 0.009). In contrast, the KPC-/OXA-48 producers were more susceptible to amikacin (*p* = 0.001) (Table 3). The highest rate of susceptibility to commonly used antimicrobials among CRE isolates was found for tigecycline (83.1%). The CRE susceptibility rate was 79.7% for colistin and 32.8% for fosfomycin. The susceptibility to fosfomycin was higher among KPC/OXA-48 producers (*p* = 0.04, Table 3). 

The susceptibility rates to the five newly approved antibiotics in decreasing order were as follows: 90.6% for plazomicin, 76.3% for eravacycline, 37.5% for cefiderocol (with the EUCAST breakpoints), 34.4% for ceftazidime/avibactam and 26.7% for meropenem/vaborbactam. If the CLSI 2021 criteria were applied, susceptibility to cefiderocol increased to 71.8% (mainly the NDM/VIM-producing isolates were susceptible (*p* = 0.0001), Table 3). The susceptibility to ceftazidime/avibactam and meropenem-vaborbactam was mainly associated with the KPC/OXA-48 producers’ group (*p* = 0.0001), except meropenem-vaborbactam–OXA-48 producers, which were resistant to it. KPC/OXA-48 producers were more frequently susceptible to ceftazidime/avibactam than MBL producers (*p* = 0.0001); a similar difference was observed regarding meropenem/vaborbactam (*p* = 0.0001) with one exception: all *bla*_OXA-48_ positive isolates were resistant to the agent. Interestingly, three VIM-1 producing *P. mirabilis* isolates were susceptible to ceftazidime/avibactam and meropenem-vaborbactam. The distribution of the MICs of tested isolates are shown in Table 4. For tigecycline, eravacycline and plazomicin, FDA breakpoints were used. By applying the EUCAST criteria for tigecycline, only 3 isolates were in the susceptible category. The lowest MIC_90_ was found for eravacycline (1.5 mg/L), followed by tigecycline and plazomicin (4 mg/L). Colistin (MIC_90_ > 16 mg/L), meropenem/vaborbactam (128 mg/L) and fosfomycin (≥256) had higher MICs_90_ values.

An extensively drug resistant phenotype was found in 35 (55%) of the tested isolates (29 *K. pneumoniae*, three *P. mirabilis*, two isolates *P. stuartii* and one *E. cloacae* complex). They were distributed in all hospitals included in the study (12 of 22 received from Sof-3, 15 of 22 received from Sof-1, 5 from PL and only three from VR). Most of them belonged to the ST11 clone (22 isolates). XDR isolates were susceptible to tigecycline in 70% (21/30), amikacin 11.4% (4/35), gentamicin 31.4% (11/35), fosfomycin 14.2% (5/35), tigecycline in 60% (21/35) and colistin 51.4% (18/35). Among the novel antimicrobials, plazomicin showed the best activity of 91.4% (32/35), followed by eravacycline at 63% (19/30), cefiderocol 51.4% (18/35), ceftazidime/avibactam 25.7% (9/35) and meropenem/vaborbactam 20% (7/35).

## 4. Discussion

The production of carbapenemases among isolates of the order *Enterobacterales* is one of the most important problems in contemporary medical practice. The plasmids carrying the carbapenemase gene often have additional genes conferring resistance to other antimicrobial groups, resulting in multidrug resistant, extensively drug resistant and, in some cases, pandrug-resistant bacteria [3]. Nowadays, we rely on limited options for treatment of problematic infections, such as colistin, tigecycline, aminoglycosides, fosfomycin and different combinations between them or with carbapenems [11,12]. Unfortunately, the use of some of these agents is accompanied by side effects like toxicity or emergence of resistance. The antimicrobial agents newly introduced into clinical practice, such as the beta-lactam/beta-lactamase inhibitor ceftazidime/avibactam and meropenem/vaborbactam combinations, eravacycline, the aminoglycoside plazomicin, and the siderophore cephalosporin cefiderocol, represent novel promising therapeutic options for infections caused by carbapenemase-producing enterobacteria [12,13]. The present study evaluated the activity of these novel agents against a collection of CRE, isolated in four Bulgarian hospitals. *K. pneumoniae* dominated among the clinical isolates. During the study period, only single carbapenem-resistant isolates of *E. cloacae* complex, *C. freundii*, *P. stuartii* and *P. mirabilis* were detected. The NDM-1 producing *K. pneumoniae* were the most common CRE isolates, often co-harboring *bla*_CTX-M-15/-3_ and/or *bla*_CMY-4_ and belonged predominantly to the ST11 clone. ST11 is known to be a successful high-risk international clone, widely disseminated in Asia(China), Europe (Greece, Czech Republic, Poland, Spain) and the USA and carrying different carbapenemase genes (*bla*_KPC_, *bla*_VIM_, *bla*_OXA-48_, *bla*_NDM_) [22,23,24,25,26,27,28,29,30]. In addition, some isolates [22] from this clone exhibited the presence of three beta-lactamase genes—one carbapenemase *bla*_NDM-1_, one ESBL *bla*_CTX-M-15_ or *bla*_CTX-M-3_, and one AmpC enzyme *bla*_CMY-4_, thus increasing the high level of resistance to many antimicrobial agents. We observed 35 (54.7%) of the isolates to be extensively drug-resistant isolates.

The KPC-2 producers, with a relative proportion of 24%, were represented by ST340 and ST258, single locus variants of ST11, and both co-producing KPC-2 and CTX-M-15/-3 ESBLs. Our previous multicenter study demonstrated that after their first appearance in 2012, KPC-2 producers dominated in Varna and Pleven in the 2015–2016 period, but have been replaced by NDM-1-producing *K. pneumoniae* since 2017 [9]. The present study confirms this trend. The *bla*_VIM-1_ gene (concomitantly with *bla*_CMY-99_ gene) was only found in three isolates of *P. mirabilis* and two *P. stuartii* isolate. *P. mirabilis* carrying both *bla*_VIM-1_ and *bla*_CMY-99_ has been reported before [31]. Our study identified for the first time CMY-99-producing *P. stuartii* in Bulgaria, thus demonstrating the potential for *bla*_CMY-99_ transfer between different bacterial species. 

The OXA-48 producers in this study were mainly associated with *K. pneumoniae* ST101, but also with *E. cloacae* ST200. ST101 is an emerging nosocomial high-risk clone associated with infections with increased mortality compared with non-ST101 infections; it has appeared sporadically in different countries [32]. The association of ST101 with OXA-48 beta-lactamase production has been reported in multiple studies from different geographical regions [33]. The *E. cloacae* complex included in this study belonged to ST200. Members of this clone are part of the *Enterobacter xianfangensis* species. ST200 *E. cloacae* complex is characteristically multidrug resistant and reported as a carrier of carbapenemases such as NDM-1 [34].

Colistin, tigecycline, aminoglycosides, and fosfomycin could be used, in case of in vitro susceptibility, in different combinations with or without carbapenems (if their MICs are below 8 mg/L) or as monotherapy [11]. Until recently, they were last-line antibiotics for the treatment of carbapenem-resistant enterobacterial infections [11]. However, their prolonged use has led to the emergence of resistance. In the last few years, colistin has been widely used to treat ventilator-acquired pneumonia, bloodstream infections, abdominal infections, and urinary tract infections caused by CRE [11,12,13]. Its use as monotherapy for infections caused by CRE is associated with a significant risk of developing drug resistance in the isolates [3,11,25]. While in the past, a majority of isolates were susceptible to colistin, allowing monotherapy or combinations [11,12,13], resistance has now emerged. In the present study, the colistin resistance (20.3%) was lower than that observed in a previous study conducted in Bulgaria (29%, with 8% heteroresistant strains). A possible reason explaining this difference is the fact that the present study was multicenter. The observed colistin resistance data in this study (20.3%) were lower than those reported for Greece (34.2%) [30], Dubai (59%) [35] and London (65.8%) [36], but higher than those in China (1.4%) [37]. In the present study, we still found low levels of susceptibility to gentamicin and amikacin (20.3–35.9%). Existing susceptibility to aminoglycosides provides clinicians an important clinical benefit for the treatment of patients with CRE infections (in case of non-severe infections) [11]. A statistically significant difference was demonstrated between the susceptibility to aminoglycosides and the types of produced carbapenemases in the current study. NDM-1/VIM-1 producers were more susceptible to amikacin (46.7%), while the KPC-2 and OXA-48 isolates were more susceptible to gentamicin (47.3%). 

**Plazomicin** is a novel, semisynthetic aminoglycoside derived from sisomicin and containing structural modifications that allow it to maintain its activity in the presence of many aminoglycoside-modifying enzymes. The agent was approved for clinical use by the FDA in 2018. The complicated urinary tract infections caused by enteric bacteria, including carbapenem-resistant ones, are indications for the use of plazomicin [38]. Many studies have shown the excellent activity of plazomicin against carbapenem-resistant *Enterobacterales* in South America, the UK, the USA [39,40] and Spain [41]. The present study confirmed these results, finding a 90.6% susceptibility rate among the Bulgarian carbapenem-resistant isolates, 70% of which were MBL producers. The observed MIC_50_/MIC_90_ values (0.5 mg/L/4 mg/L) in the present study were low. We found no correlation between plazomicin susceptibility and the type of carbapenemase produced. In both groups of carbapenem-resistant isolates, susceptibility ranged between 90% and 95%. Similarly, the multicenter study in US hospitals in 2016–2017, conducted by Castanheira et al., reported the levels of plazomicin susceptibility among carbapenem-resistant *Enterobacterales* to be between 95.9–96.7% [42]. A study in Greece [30] also reported results similar to ours, with MIC_plazomicin_ ≤ 1.5 mg/L for 94% of the tested isolates. The results obtained on the activity of this antimicrobial agent identify it as a very good alternative in cases of problematic UTIs. Potential limitations for its use are the possible side effects typical for all aminoglycosides, such as ototoxicity, nephrotoxicity and neuromuscular blockade [43]. So far, EUCAST has no published breakpoints for plazomicin [17]. The comparison between the gradient diffusion method (MICstrip for plazomicin) and the standard broth microdilution method shows excellent agreement (99%) [44]. Unfortunately, to date, plazomicin has not been registered for use in Europe [12]. The importance of this agent has grown since the publication of some reports on its use in the treatment of bacterial pneumonia and bloodstream infections. Denervaud-Tendon et al. (2017) reported that 28-day all-cause mortality or clinically significant complications were found in 24% of the patients receiving plazomicin, compared with 50% among patients receiving colistin in combination with meropenem or tigecycline for 7 to 14 days of treatment [45]. The resistance to plazomicin is associated with 16S methyl transferases, which confer higher levels of resistance to all aminoglycosides. An association of 16S rRNA methyltransferases and different carbapenemases (OXA-48, NDM) in *Enterobacterales* has been increasingly reported [46]. In addition, *bla*_CTX-M-3_ has been identified very often on IncL/M plasmids, also carrying genes coding *armA* methylases [47]_._ In the present study, we observed a lower prevalence of *bla*_CTX-M-3_. Based on this result, we can presume a lower prevalence of methylases (ArmA), which would explain the relative susceptibility to gentamicin/amikacin and the very high susceptibility to plazomicin. 

**Tigecycline** and **ravacycline** are the agents from the tetracycline group that were tested in the present study. **Tigecycline** is a representative of the glycylcyclines and is among the few effective agents for the treatment of infections caused by CRE. However, the lack of breakpoints for tigecycline in EUCAST for bacterial species other than *E. coli* and *C. koseri* is problematic. The present study showed that the MIC_50/90_ of tigecycline was 1 mg/L and 4 mg/L, respectively. If FDA recommendations are used, the tigecycline susceptibility in the studied group of isolates is 83.1%. If we interpret these values by EUCAST breakpoints (for *E. coli*, ≤0.5 mg/L susceptible category) [17], only three isolates can be determined to be susceptible, which indicates the need to harmonize breakpoints between the two standards. Our results were similar to the results obtained in Singapore (89.6% susceptibility with FDA criteria) [48] and Greece (80.5% susceptibility rate) [30]. The EUCAST breakpoints for *E. coli* are supposed to be more relevant, as some researchers reported therapeutic success if MICs were below 0.5 mg/L [4,49]. In general, tigecycline is an antibiotic approved for the treatment of complicated skin and soft tissue infections, complicated intra-abdominal infections, and community-acquired bacterial pneumonia in individuals aged 18 years and over, with the age range changed to 8 years and over in the UK [50], but if CRE infections are the target, it should be the last choice and should not be used for bloodstream and hospital-aquired and ventilator-associated pneumonia. If necessary, in patients with pneumonia, clinicians may use high-dose tigecycline [49]. It is worth mentioning that tigecycline has a bacteriostatic effect on most enterobacteria, and in recent years, a black box warning has been reported, as, similarly to colistin, this antimicrobial agent can lead to increased mortality rates if used inappropriately [4].

**Eravacycline** is a novel fluorocycline that was introduced to solve the problem with tetracycline resistance mechanisms among Gram-negative bacteria (except *P. aeruginosa*) such as efflux pumps and ribosomal protection proteins [12,51]. The isolates in the present study (excluding *P. mirabilis* and *P. stuartii* isolates) demonstrated good susceptibility to eravacycline with a MIC_50_ of 0.38 mg/L and MIC_90_ of 1.5 mg/L. These MICs were two to three times lower than those of tigecycline. However, interestingly, the detected susceptibility rate for eravacycline (76.3%) was lower compared with that for tigecycline (83.1%), which could be explained with the current FDA breakpoints applied. Other authors have also reported similar findings. Teo et al. reported 53.4% susceptibility to eravacycline and 89.6% to tigecycline [48]. Higher levels of susceptibility to tigecycline and eravacycline (89% and 85.7% respectively) have been reported by authors from China [52]. Similar to our findings are the results reported by Maraki et al., who also detected higher susceptibility to tigecycline (86%) and lower to eravacycline (60%) among carbapenem-resistant *Enterobacterales* [30]. A recent study from Poland demonstrated good in vitro susceptibility of MBL- and KPC-producing isolates to eravacycline in 59% and 73% of the isolates, respectively [53]. Our study confirmed these findings and demonstrated higher (89.5%) susceptibility to eravacycline in the KPC/OXA-48 group than that among the NDM-1/VIM-1-producers (70%). Interestingly, for tigecycline, there was no difference between the two groups. However, eravacycline has been approved only for the treatment of complicated intra-abdominal infections, which significantly limits its use [52]. Some authors have discussed the positive outcome of its usage in complicated UTIs, probably due to its effect on the biofilms produced by uropathogenic *E. coli* [54]. 

New antibacterial agents already introduced into clinical practice for the treatment of CRE infections include some combinations of beta-lactam agent with a beta-lactamase inhibitor such as **ceftazidime/avibactam** and **meropenem/vaborbactam**. They are the first choice for severe CRE infections if in vitro susceptibility is confirmed [49]. Registered in 2015, the main indications for use of ceftazidime/avibactam include complicated intra-abdominal infections, complicated urinary tract infections, and hospital-acquired and ventilator-associated pneumonia [13]. Avibactam is a novel non-β-lactam inhibitor, with a broad spectrum of activity including class A, C and some class D β-lactamases. This β-lactamase spectrum defines the ceftazidime/avibactam combination as an alternative in cases of difficult-to-treat infections, excluding those caused by MBL producers. Vaborbactam is a non-antibiotic cyclic boronic acid inhibitor that can bind reversibly with meropenem [13]. The combination improves the stability of meropenem against class A and C beta-lactamases but is inactive toward class B and D enzymes. The two combined formulations affect a similar spectrum of infections. A low proportion of carbapenem-resistant isolates susceptible to both combinations—34.4% for ceftazidime/avibactam and 26.7% for meropenem/vaborbactam—was found in the present study. These rates were much lower than those reported in Greece (79.9% susceptibility to ceftazidime/avibactam and 76.3% to meropenem/vaborbactam), which may be explained by the higher relative proportion of KPC producers in the Greek study [30]. In contrast, our collection was dominated by metallo-carbapenemase producers. The results from the present study showed that ceftazidime/avibactam has activity against KPC/OXA-48 producers but not against NDM-1 producing isolates. Meropenem/vaborbactam has the same pattern, except that it has no activity against OXA-48 producers. Our findings are consistent with previous studies, reporting good in vitro activity of these antibiotics against carbapenemase-producing but MBL-negative *K. pneumoniae* isolates [55,56]. Interestingly, in the present study, three *bla*_VIM-1_ positive isolates demonstrated susceptibility to both agents. We can assume that this result could be related to the level of *bla*_VIM-1_ gene expression. As ceftazidime/avibactam is not effective against metallo-carbapenemases, many authors have suggested an addition of aztreonam to ceftazidime/avibactam to increase the efficacy of the combination [49,57]. 

Usually, fosfomycin, a representative of the epoxide class of antibiotics, is part of the standard susceptibility testing in cases of infections associated with carbapenemase-producing *Enterobacterales* and possibly part of the combination therapy, as it may impact CRE [11]. In this study, a relatively low proportion of fosfomycin-susceptible isolates were found (32.8%), which raises the question of its usefulness in combined therapeutic regimens.

**Cefiderocol** is the first catechol-substituted siderophore cephalosporin with a unique mechanism of bacterial cell entry [58]. It is stable to hydrolysis by serine class A, class B, and MBL enzymes. Some reports also showed its stability to OXA-48 enzymes [12]. In 2019, cefiderocol was approved by the FDA for complicated urinary tract infections and in 2020 for hospital-acquired pneumonia/ventilator-associated pneumonia caused by susceptible pathogens, as well as for adults with limited treatment options [12]. According to ESMID recommendations, cefiderocol could be used in monotherapy for infections caused by MBL-producing enterobacteria or *Enterobacterales* isolates resistant to ceftazidime/avibactam or meropenem/vaborbactam [49]. Authors from Spain and Portugal reported excellent susceptibility rates to cefiderocol—between 94.5% and 99.5% according to the used guidelines (EUCAST and CLSI, respectively) [59]. A large multicenter Italian study demonstrated good clinical effectiveness of this novel agent. The authors did not observe a significant difference in the mortality rates between the patient group that received cefiderocol as a monotherapy and the group that received a standard combined therapy (33% versus 40%) [60]. A study conducted by Portsmouth et al. compared cefiderocol and imipenem/cilastatin for the treatment of complicated urinary tract infections and reported a significant treatment difference in favor of cefiderocol [61].

Unfortunately, cefiderocol-resistant isolates have already been reported, and among them, the NDM producers prevail [62]. In the present study, we observed very low susceptibility rates when the EUCAST criteria were applied (37.5%) and respectively higher susceptibility rates when the results were interpreted according to CLSI guidelines—71.8% susceptibility. Our results are consistent with the study of Isler et al. [63], who reported 81% and 12% resistance, respectively, for cefiderocol among NDM producers using the EUCAST and CLSI interpretation criteria. Similar results were reported by other authors for *A baumanii* [64]. This discrepancy, when the disk-diffusion method was used, is very large and requires breakpoint harmonization, as the resistance may have a significant impact on the patient’s outcome. An important finding from the current study was the fact that MBL producers were more susceptible to cefiderocol than KPC/OXA-48 (88.9% versus 31.6%, respectively, *p* = 0.0001). On the basis of this finding, we can suppose that after in vitro confirmation, cefiderocol can be used for infections caused by CRE/MBL isolates. This corresponds to ESCMID recommendations, as cefiderocol is currently recommended for the treatment of severe infections when the strains are MBL producers or/and are resistant to ceftazidime/avibactam and meropenem/vaborbactam [49]. The low rate of susceptible isolates (among our isolates) could be due to combination of two or three beta-lactamases in one isolate (one carbapenemase or/and one AmpC or/and one ESBL). Another probable explanation and a possible source of errors is the methodology of cefiderocol susceptibility testing. The microdilution method in iron-depleted broth is the gold standard [17]. Some authors have suggested the disk diffusion method for screening purposes, and the CRE isolates should be retested using broth microdilution with iron-depleted broth to assess the final categorization [65].

A **limitation** of our work is the small number of isolates. Further investigation can enlarge the number of isolates and encompass some other promising antimicrobials such as aztreonam/avibactam.

## 5. Conclusions

To the best of our knowledge, this is the first study in Bulgaria to report the susceptibility rates of carbapenem-resistant enterobacteria to five novel antimicrobial agents and their association with particular genotypes. Sixty-four carbapenemase producers were collected in a period of one year from four Bulgarian hospitals, mainly including *K. pneumoniae* (89% of the isolates). The main genotype was *bla*_NDM-1_ (61%), followed by *bla*_KPC-2_ (23%) and single isolates *bla*_VIM-1_ and *bla*_OXA-48_; thus, MBL producers were around 70%. The most common MLST type was *K. pneumoniae* ST11 (57.8%), followed by ST340 (12.5%), ST258 (6.3%) and ST101 (6.3%). The isolates were highly resistant to standard-group antibiotics; however, they were susceptible to tigecycline (83.1%) and colistin (79.7%), which can be used in combination schemes when appropriate. Among the newly approved compounds, plazomicin (90.6%) and eravacycline (76.3%) exhibited the best activity, but they have been approved for the restricted types of infections. In addition, plazomicin has not yet been approved for use in Europe. The susceptibility to ceftazidime/avibactam and meropenem/vaborbactam was restricted to 34.4% and 27.6%, respectively, of our strains, which was associated with the MBL producer prevalence in our collection. Both combinations can be used for KPC producers, and ceftazidime/avibactam for OXA-48 producers. An important finding was the susceptibility of our VIM-1 producing isolates to ceftazidime/avibactam. For cefiderocol, a big discrepancy has been observed between the susceptibility rates (37.5% by the disk-diffusion method) according to EUCAST breakpoints and 71.8% according to CLSI breakpoints, which shows the need for harmonization of the breakpoints. Interestingly, around 90% of our MBL producers were susceptible to cefiderocol using the CLSI breakpoints, which allows its use in case of severe CRE infections after in vitro confirmation of susceptibility. However, most of the newly introduced antibiotics are relatively expensive and are still not licensed in many countries. Due to the limited options for therapy, infection control measures and antibiotic policy remain very important. We hope that our data will contribute to appropriate tailored treatment of patients with CRE infections.

## Figures and Tables

**Table 1:** Distribution of carbapenemase-producing enterobacterial isolates by center and type of carbapenemase genes detected.

Hospital Designation	Hospital, City	Beds (*n*)	Bacterial Isolates (*n*)				
			*bla* Genes				Total
			*bla*_KPC-2_	*bla*_NDM-1_	*bla*_VIM-1_	*bla*_OXA-48_	
Sof-1	Medical institute, Ministry of the Interior, Sofia	310	3	15	4		22
Sof-3	University Multiprofile Hospital for active treatment (UMHAT) “Ivan Rilski”, Sofia	395	1	15		3	19
VR	UMHAT “St Marina”, Varna	1250	11	4	0	1	16
PL	UMHAT “Georgi Stranski”, Pleven	1000		5	1	1	7
Total			15	39	5	5	64

Abbreviations: *n*—number.

**Table 2:** Detected bacterial genes of carbapenemases, ESBLs and AmpC enzymes in 5 bacterial species and their clonal distribution.

	Bacterial Species	*K. pneumoniae* *n* = 54 ST Types	*P. mirabilis* *n* = 5	*C. freundii* *n* = 2	*E. cloacae* *n* = 1	*P. stuartii* *n* = 2
Genotypes						
*bla*_NDM-1_ + *bla*_CTX-M-3/-15_ + *bla*_CMY-4_		ST11*_n_*_=20_ ST307_*n*__=2_				
*bla*_NDM-1_ + *bla*_CTX-M-3/-15_		ST11*_n_*_=12_				
*bla*_NDM-1_ + *bla*_CMY-4_		ST11*_n_*_=2_				
*bla*_NDM-1_		ST11*_n_*_=3_				
*bla*_KPC-2_		ST258*_n_*_=4_				
*bla*_KPC-2_ + *bla*_CTX-M-15/-3_		ST340*_n_*_=8_ ST34_*n*__=1_		2		
*bla*_OXA-48_ + *bla*_CTX-M-15_		ST101*_n_*_=4_			1 (ST200)	
*bla*_VIM-1_ + *bla*_CMY-99_		-	3			2

Abbreviations: *n*—number isolates.

**Table 3:** Susceptibility testing of 64 carbapenemase-producing bacterial isolates to set of antibacterial agents.

Antimicrobial Agents (*n* *)	Result Interpretation			KPC-/OXA-48 Producers (*n* = 19)	NDM-/VIM Producers (*n* = 45)	*p* Value
	R %	I %	S %	S% (number)	S % (number)	
**Disk diffusion method**						
Amoxicillin/Clavulanic acid, *n* = 64	100.0	0	0	0	0	
Piperacillin/Tazobactam, *n* = 64	100.0	0	0	0	0	
Cefotaxime, *n* = 64	100.0	0	0	0	0	
Ceftazidime, *n* = 64	100.0	0	0	0	0	
Cefepime, *n* = 64	100.0	0	0	0	0	
Cefoxitin, *n* = 64	96.9	0	3.1	10.5% (2)	0	
Imipenem, *n* = 64	100.0	0	0	0	0	
Meropenem, *n* = 64	100.0	0	0	0	0	
Tobramycin, *n* = 64	100.0	0	0	0	0	
Gentamicin, *n* = 64	64.1	0	35.9	**10.5% (2)**	**46.7% (21)**	**0.009**
Amikacin, *n* = 64	79.7	0	20.3	**47.3% (9)**	**8.9% (4)**	**0.001**
Ciprofloxacin, *n* = 64	100.0	0	0	0	0	
Levofloxacin, *n* = 64	100.0	0	0	0	0	
Trimethoprim/Sulfamethoxazole, *n* = 64	100.0	0	0	0	0	
Ceftazidime/avibactam, *n* = 64	65.6	0	34.4	**94.7% (18)**	**6.7% (3)**	**0.0001**
Cefiderocol, *n* = 64/EUCAST	62.5	0	37.5	26.3% (5)	42.2% (19)	
Cefiderocol, *n* = 64/CLSI	10.9	17.1	71.8	**31.6% (6)**	**88.9% (40)**	**0.0001**
**MIC determination**						
Fosfomycin, *n* = 64	67.0	0	32.8	**52.6% (10)**	**24.4% (11)**	**0.04**
Colistin **, *n* = 59	20.3	0	79.7	84.2% (16)	77.5% (31)	
Tigecycline **, *n* = 59	16.9	0	83.1	84.2% (16)	82.5% (33)	
Eravacycline **, *n* = 59	23.7	0	76.3	**89.5% (17)**	**70.0% (28)**	**0.03**
Plazomicin, *n* = 64	6.4	3.0	90.6	89.5% (17)	91.1% (41)	
Meropenem/vaborbactam, *n* = 64	73.3	0	26.7%	**73.7% (14)**	**6.7% (3)**	**0.0001**
Imipenem, *n* = 64	100.0	0	0	0	0	
Meropenem, *n* = 64	95.3	4.7	0	15.8% (3)	0	

Legend: R, resistance; I, intermediate susceptibility; S, susceptibility; *n* *, number of tested isolates; ** *P. mirabilis* and *P. stuartii* were excluded from the testing, the percentages in bold referred to statistically significant differences (when *p* was <0.05)

**Table 4:** Minimal inhibitory concentrations of tested antibiotics.

Antibiotics	MIC mg/L																			
	≤0.25	0.38	0.5	0.75	1	1.5	2	4	6	8	12	16	24	32	48	64	128	>256	MIC_50_	MIC_90_
Tigecycline *		2	1	10	23	11	2	10											1	4
Eravacycline *	17	17	11	4	2	6	1												0.38	1.5
**Fosfomycin**							** 2 **	** 5 **		** 5 **				** 9 **	4	11		28	64	≥256
**Plazomicin**	6	19	25	4			2	2										6	0.5	4
**Meropenem/** **Vaborbactam ^1^**	** 9 **		** 3 **	** 2 **	** 1 **	** 2 **					5	4	7	5	5	8	5	6	24	128
Colistin *	** 34 **		** 8 **		** 4 **		** 1 **	2		3		7 ^χ^							0.25	≥16

Legend: *, three *P. mirabilis* and 2 *P. stuartii* were excluded from the testing, χ–this number is for ≥16 mg/L; numbers in bold are in susceptible category according to EUCAST, 2023; underlined numbers are in susceptible category according to FDA recommendations; ^1^-fixed concentration of 8 mg/L vaborbactam has been used.

## Data Availability

All data generated for this study are contained within the article.
